# No associations between fruit and vegetable consumption and pancreatic cancer risk: a meta-analysis of prospective studies

**DOI:** 10.18632/oncotarget.23128

**Published:** 2017-12-08

**Authors:** Zhanwei Zhao, Pengfei Yu, Xiangying Feng, Zifang Yin, Shiqi Wang, Zhaoyan Qiu, Qingchuan Zhao

**Affiliations:** ^1^ Department of Surgery, Navy General Hospital of PLA, Beijing, China; ^2^ Xijing Hospital of Digestive Diseases, The Fourth Military Medical University, Xi'an, China; ^3^ Shaanxi Maternal and Child Health Hospital, Shaanxi Province, Xi'an, China; ^4^ The General Hospital of The People’s Liberation Army, Department of General Surgery, Beijing, China

**Keywords:** meta-analysis, fruit, vegetable, pancreatic cancer, risk

## Abstract

The associations between fruit and vegetable consumption and pancreatic cancer risk are inconclusive. We conducted a meta-analysis of prospective studies to investigate the associations. The search was conducted systemically using the PubMed and EMBASE databases up to March 2017. Relative risks and 95% confidence intervals for the highest versus lowest consumption and dose-response analyses were assessed. Subtype and subgroup analyses were performed. Twelve studies were eligible. The summary relative risks of the highest versus lowest consumption were 0.95 (0.80–1.12) for total fruits and vegetables without heterogeneity (*I*^2^ = 0%, *P =* 0.44), 0.96 (0.82–1.12) for fruits without low heterogeneity (*I*^2^ = 37%, *P =* 0.12) and 0.94 (0.84–1.06) for vegetables with low heterogeneity (*I*^2^ = 9%, *P* ***=*** 0.36). Dose-response analyses also showed no significantly inverse associations for each 100 g/day increase; the summary relative risks were 1.00 (0.98–1.02) for total fruits and vegetables, 1.01 (0.97–1.05) for fruits and 1.00 (0.97–1.03) for vegetables. The results of subtype analyses were consistent with the fruit and vegetable analyses; the relative risks were 0.97 (0.80–1.17) for citrus fruit without low heterogeneity (*I*^2^ = 39%, *P =* 0.15) and 0.89 (0.76–1.05) for cruciferous vegetables without low heterogeneity (*I*^2^ = 14%, *P* ***=*** 0.32). In conclusion, this meta-analysis does not support significant associations between fruit and vegetable consumption and pancreatic cancer risk.

## INTRODUCTION

Pancreatic cancer (PC) is a highly sinister disease with an extremely poor prognosis and a five-year survival rate of less than 5% [1]. PC is the fourth leading cause of cancer-related death for males and females in the United States with the least improvement in survival during the past 30 years [1, 2]. Although some dietary factors have been considered to be associated with PC risk, no dietary factors have been convincingly established for PC as reported by the Continuous Update Project of the World Cancer Research Fund (WCRF) in 2012 [3]. Consumption of fruit and vegetable was often considered to be protective against PC. However, most of the data are derived from case-control studies, which may be subject to inaccurate measurements of dietary consumption and recall bias. Additionally, citrus and cruciferous vegetables, as important fruits and vegetables, were considered to be protective against cancers [4–6], but there have been no further investigations in PC. The Continuous Update Project of WCRF in 2012 (which is based on prospective studies published through 2011) reported that the evidence for fruit and vegetable consumption in reducing PC risk was “limited – no conclusion” because of the inconsistent data. Wu et al [7] carried out a meta-analysis and found that fruit and vegetable intake is associated inversely with pancreatic cancer risk. However, the authors acknowledged that there was significant heterogeneity in the combined evaluations (cohort studies and case-control studies) and study design may play a key role for their findings (significant inverse associations were observed in the analysis of case-control studies but not cohort studies). Koushik et al [8] carried out a pooled analysis of cohort studies and found that during adulthood, fruit and vegetable intake is not associated with a reduced pancreatic cancer risk. Nevertheless, the included studies were published up to 2005 in Koushik et al study [8] and high-quality studies have appeared during the last 11 years (approximately).

Therefore, in consideration of the large burden of PC worldwide and the controversial evidence, we conducted an updated meta-analysis of prospective studies based on a quantitative amalgamation of the eligible data with the following objectives: (1) to gain a better understanding of the associations of fruit and vegetable consumption with PC risk; (2) to further examine the associations according to subtype analyses for citrus fruit and cruciferous vegetables and subgroup analyses for gender and other factors, including geographic area, sample size, publication year, periods of follow-up and main adjustments; and (3) to further evaluate the dose-response associations between fruit and vegetable consumption and PC risk.

## RESULTS

### Literature selection, study characteristics and quality scores

**Figure 1:**
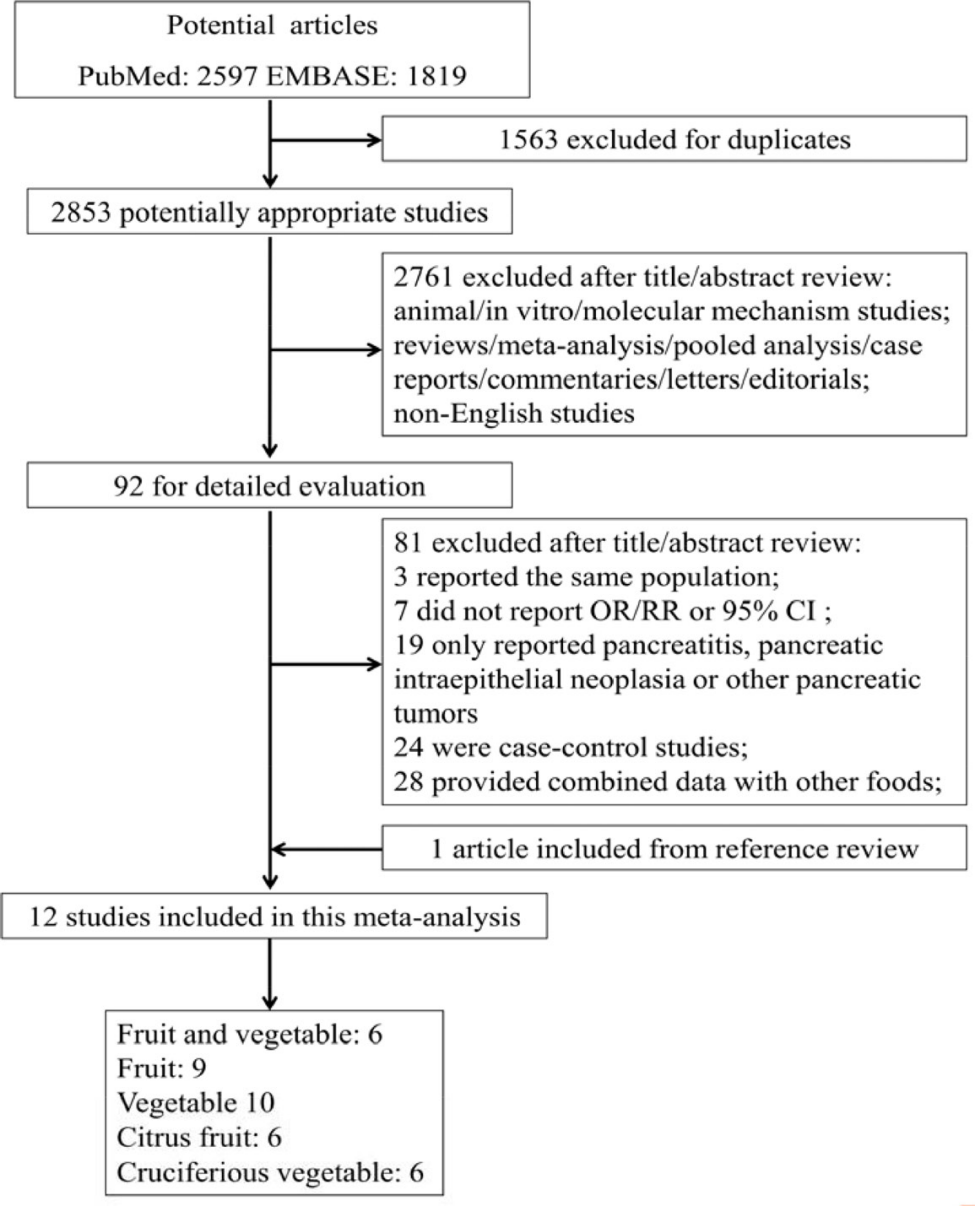
Flowchart of the processs for the identification of relevant studies

Figure 1 shows the flowchart of the search strategy for selecting the eligible studies. A total of 4416 studies were initially identified for this meta-analysis; 2853 studies were selected for further consideration after excluding 1163 studies for duplication. Of the 2853 identified, 2761 studies were excluded after reviewing the titles and abstracts, and 81 studies were further excluded after reviewing the full-text article. Finally, 12 studies met the eligibility criteria after including 1 study from the reference review. The range of the quality scores was 6–9 for PC and the mean study quality score was 7.33 (Table 1). The 12 selected studies were from 13 countries in North America, Europe and Asia with 1,263,396 participants and 3,300 cases (Table 1). The references and exclusion reasons for the excluded studies were listed in Table 2.

**Table 1:** Baseline characteristics of studies investigating fruit and vegetable consumption and pancreatic cancer risk

First author, year, country or region	Cases/participants	Study population	Study period	Dietary assessment	Exposure categories	Type, RR (95% CI)	Controlled variables	Quality score
Shibata 1994 USA [15]	63/13979	M & F	1981–1990	FFQ-59	Tertile	Fruit, 0.89 (0.49–1.62) Vegetable, 0.82 (0.44–1.51)	Age, sex, smoking	6
Stolzenberg-solomon 2002 Finland [16]	163/27111	M	1985–1997	FFQ-276	Quintile	Fruit and vegetable 0.74 (0.46–1.20)	age, smoking, energy intake	6
Inoue 2003 Japan (nested case-control) [26]	200/2000	M & F	1988–1999	FFQ-NS	Less versus every day	Vegetable, 0.71 (0.51–0.99)	age, gender, family history of pancreatic cancer, history of diabetes, physical exercise, bowel habits and alcohol	7
Larsson 2006 Sweden [17]	135/81922	M & F	1998–2004	FFQ-96	Quartile	Fruit and vegetable 1.13 (0.66–1.94) Fruit, 1.10 (0.64–1.88) Vegetable, 1.08 (0.63–1.85)	age, sex, education, BMI, physical activity, smoking, history of diabetes, multivitamin supplement use, energy intake, alcohol	7
Nothlings^1^ 2007 USA [27]	434/162150	M & F	1993–2002	FFQ-NS	Quartile	Fruit, 1.42 (1.05–1.93)	age, sex, ethnicity, family history of pancreatic cancer, smoking, intakes of red meat and processed meat, energy intake and BMI	8
Nothlings^2^ 2007 USA [28]	529/183522	M & F	1993–2002	FFQ-180	Quintile	Vegetable, 0.86 (0.65–1.14)	age, sex, ethnicity, history of diabetes, family history of pancreatic cancer, smoking, intakes of red meat and processed meat, energy intake and BMI	8
Bobe 2008 Finland [29]	306/27111	M	1985–2004	FFQ-276	Quintile	Fruit, 0.95 (0.67–1.34) Vegetable, 0.78 (0.54–1.12)	age, smoking, history of diabetes and energy-adjusted saturated fat intake	7
Vrieling 2009 Europe [30]	555/478400	M & F	1991–2000	FFQ-NS	Quartile	Fruit and vegetable, 0.92 (0.68–1.25) Fruit, 1.02 (0.77–1.36) Vegetable, 0.99 (0.73–1.33)	age, sex, energy, BMI, history of diabetes, smoking	8
George 2009 USA [31]	713/288109	M	1995–2003	FFQ-124	Quintile	Fruit, 0.73 (0.57–0.95) Vegetable, 1.03 (0.81–1.32)	age, smoking, energy intake, BMI, alcohol, physical activity, education, race, marital status, family history of cancers and fruit intake	8
Inoue-choi 2011 USA [32]	256/34642	F	1991–2007	FFQ-42	Quintile	Fruit and vegetable, 1.18 (0.79–1.77) Fruit, 0.98 (0.64–1.50) Vegetable, 1.21 (0.81–1.80)	age, race, alcohol, education, smoking and physical activity	7
Heinen 2011 Netherlands [33]	406/120852	M & F	1986–2002	FFQ-150	Quintile	Fruit and vegetable, 0.89 (0.64–1.24) Fruit, 0.90 (0.66–1.24) Vegetable, 1.23 (0.86–1.75)	age, sex, smoking, BMI, history of diabetes, family history of pancreatic cancer, energy intake, red meat, coffee and alcohol	9
Shigihara 2014 Japan [18]	137/32859	M & F	1994–2005	FFQ-40	Tertile	Fruit and vegetable, 0.57 (0.39–1.11) Fruit, 0.64 (0.32–1.20) Vegetable, 0.67 (0.33–1.35)	age, BMI, family history of cancer, history of diabetes, smoking, alcohol, physical activity, education, marital status, job status, meat and energy intake	7

FFQ: food frequency questionnaire (food items); NS: not specified; BMI: body mass index; M: males; F: females.

**Table 2:** Exclusion table for meta-analysis of fruit and vegetable consumption and pancreatic cancer risk

Excluded studies	Country	Study design	Study population	Exposure type	Exclusion reason
Mills 1988 [34]	USA	Prospective cohort	M & F	Citrus fruit	Mortality
Coughlin 2000 [35]	USA	Prospective cohort	M & F	Citrus fruit Vegetable	Mortality Mortality
Appleby 2002 [36]	UK	Prospective cohort	M & F	Fruit	Mortality
Stolzenberg-solomon 2002 [16]	Finland	Prospective cohort	M	Fruit Citrus fruit Vegetable	Superseded by Bobe 2008 Superseded by Bobe 2008 Superseded by Bobe 2008
Sauvaget 2003 [37]	Japan	Prospective cohort	M & F	Fruit	Mortality
Khan 2004 [38]	Japan	Prospective cohort	M & F	Fruit	Mortality
Lin 2006 [39]	Japan	Prospective cohort	M & F	Fruit Citrus fruit Vegetable Cruciferous vegetable	Mortality Mortality Mortality Mortality

M: males; F: females.

### The association between consumption of fruits and vegetables with PC risk

#### Highest vs lowest consumption

**Figure 2:**
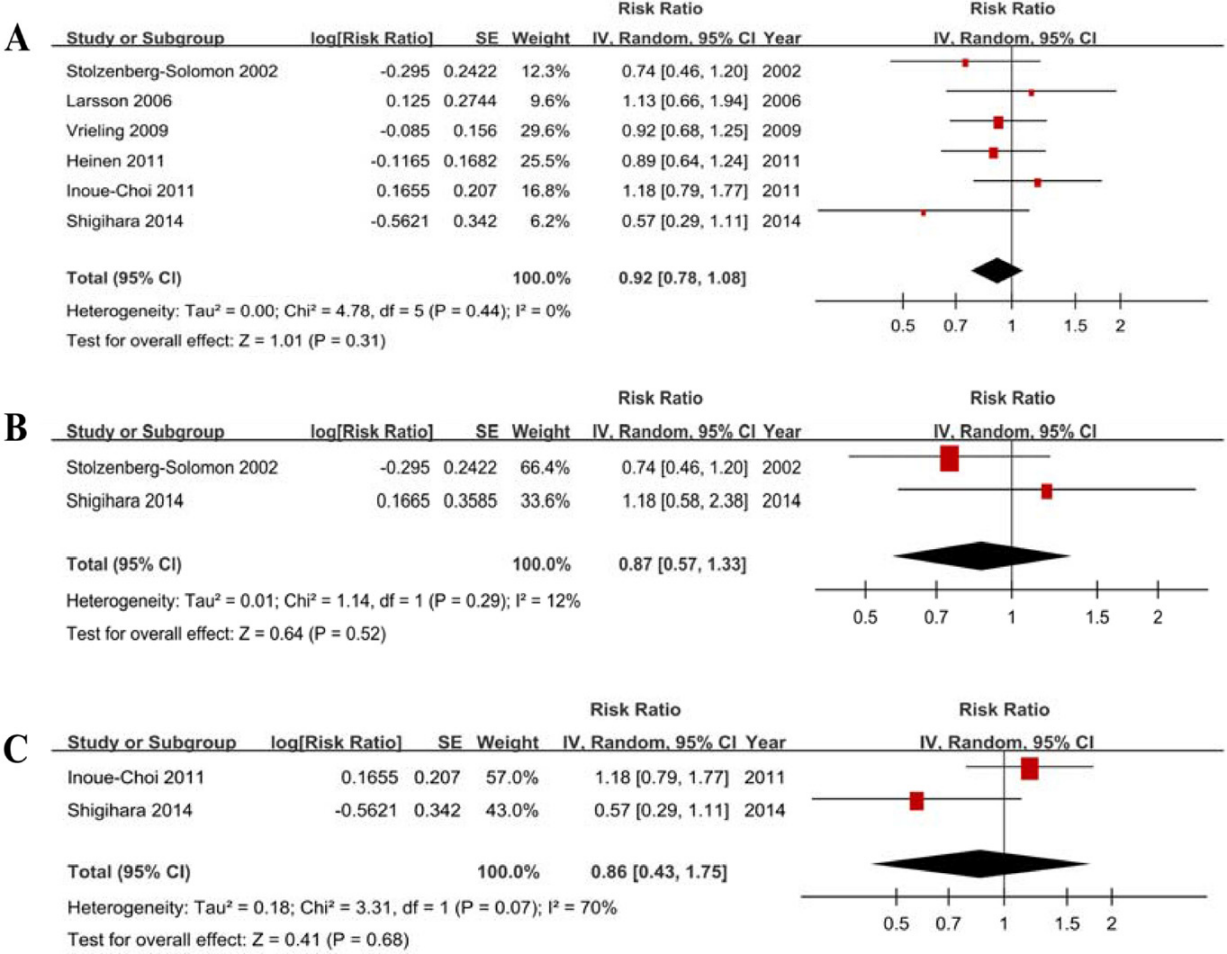
Forest plots of fruits and vegetables consumption (highest vs lowest category) and PC risk (**A**) Total estimate. (**B**) In men. (**C**) In women.

Six studies showed the results for the highest vs lowest consumption. A random-effects model yielded the results (Figure 2A) that fruit and vegetable consumption was not associated with PC risk (RR = 0.92, 95% CI = 0.78–1.08, *P* = 0.31) without heterogeneity (*P* = 0.44, *I*^*2*^ = 0%) (Table 3).

**Table 3:** Subgroup analyses of fruit and vegetable consumption and pancreatic cancer risk

Subgroups	Fruit and vegetable								Fruit									Vegetable			
	*n*	RR (95% CI)	*P*_o_	*P*_s_	*I*_s_^2^	*I*_h_^2^	*n*	RR (95% CI)		*P*_o_	*P*_s_	*I*_s_^2^	*I*_h_^2^	*n*	RR (95% CI)			*P*_o_	*P*_s_	*I*_s_^2^	*I*_h_^2^
**All studies**	6	0.92 (0.78–1.08)	.31	.44	0		9	0.96 (0.82–1.12)		.57	.12	37		10	0.94 (0.84–1.06)			.32	.36	9	
**Geographic area** **Europe** **America** **Asia**	4 1 1	0.90 (0.75–1.09) 1.18 (0.79–1.77) 0.57 (0.29–1.11)	.29 .42 .01	.72 N N	0 N N	41	4 4 1	0.97 (0.82–1.16) 0.98 (0.69–1.39) 0.64 (0.34–1.20)		.76 .91 .17	.91 .01 N	0 72 N	0	4 4 2	1.00 (0.83–1.20) 0.98 (0.83–1.15) **0.70 (0.52–0.95)**			.98 .81 **.02**	.37 .49 .88	6 0 0	53
**Sample size** **< 200** **≥ 200**	3 3	0.81 (0.56–1.16) 0.96 (0.79–1.17)	.25 .71	.26 .53	25 0	0	3 6	0.88 (0.63–1.23) 0.97 (0.81–1.18)		.46 .79	.44 .05	0 54	0	3 7	0.88 (0.62–1.24) 0.98 (0.83–1.15)			.46 .48	.55 .20	0 30	0
**Publication year** **Before 2010** **2010 or later**	3 3	0.91 (0.72–1.15) 0.91 (0.65–1.27)	.42 .57	.51 .18	0 42	0	6 3	0.99 (0.80–1.23) 0.88 (0.70–1.12)		.96 .29	.05 .54	55 0	0	7 3	0.90 (0.79–1.02) 0.94 (0.84–1.48)			.09 .45	.58 .29	0 19	9
**Follow-up (year)** **< 10** **≥ 10**	2 4	0.97 (0.74–1.26) 0.87 (0.68–1.13)	.80 .31	.51 .25	0 27	0	5 4	1.01 (0.77–1.31) 0.90 (0.74–1.10)		.97 .30	.03 .72	64 0	0	5 5	0.96 (0.83–1.11) 0.91 (0.70–1.18)			.60 .49	.85 .08	0 52	0
**Adjustments Smoking** **Yes** **No**	6 0	0.92 (0.78–1.08)	.31	.44	0		9 0	0.96 (0.82–1.12)		.57	.12	37		9 1	0.98 (0.87–1.10) 0.71 (0.51–0.99)			.70 .04	.57 N	0 N	9
**Alcohol** **Yes** **No**	4 2	0.95 (0.74–1.24) 0.86 (0.67–1.12)	.73 .26	.27 .47	23 0	0	5 4	**0.84 (0.71–0.99)** 1.09 (0.89–1.34)		**.04** .40	.54 .25	0 27	73.9	6 4	0.88 (0.74–1.04) 0.99 (0.81–1.20)			.14 .91	.78 .17	0 35	0
**BMI** **Yes** **No**	4 2	0.90 (0.74–1.09) 0.92 (0.61–1.50)	.28 .84	.47 .15	0 52	0	6 3	0.96 (0.76–1.20) 0.95 (0.74–1.21)		.70 .68	.03 .97	61 0	0	6 4	0.99 (0.87–1.14) 0.85 (0.67–1.09)			.90 .20	.58 .22	0 32	11
**Diabetes** **Yes** **No**	4 2	0.90 (0.74–1.09) 0.95 (0.61–1.50)	.28 .84	.47 .15	0 52	0	6 3	1.03 (0.85–1.23) 0.80 (0.65–0.99)		.79 .04	.20 .49	32 0	67.3	7 3	0.90 (0.77–1.04) 1.05 (0.86–1.28)			.15 .65	.31 .57	16 0	33
**Family history of PC** **Yes** **No**	2 4	0.79 (0.53–1.16) 0.97 (0.79–1.18)	.23 .76	.24 .47	27 0	0	4 5	0.91 (0.64–1.28) 0.99 (0.83–1.18)		.59 .92	< .01.98	75 0	0	4 6	0.98 (0.82–1.18) 0.90 (0.76–1.07)			.85 .24	.30 .35	19 11	0

BMI: body mass index. *P*_o_: test for over effect. *P*_s_: *P* value for heterogeneity within each subgroup. *I*_s_^*2*^: *I*^*2*^ (%) value for heterogeneity within each subgroup. *I*_h_^*2*^: *I*^*2*^ (%) value for heterogeneity between subgroups. N: not applicable. Bold text indicates statistical significance.

#### Dose-response analysis

Five studies were included, and the RR per 100 g/d increase in fruit and vegetable consumption was 1.00 (0.98–1.02) without heterogeneity (*P* = 0.63, *I*^*2*^ = 0%). We further checked for nonlinearity of the dose-response relationship between fruit and vegetable consumption and PC risk, and there was no evidence of a potential nonlinear relationship (*P*_nonlinearity_ = 0.56).

#### Heterogeneity

There was no significant heterogeneity (*P* = 0.44, *I*^*2*^ = 0%) of the included studies. Subgroup analyses showed that the differences in the RRs were not significant for the geographic area, sample size, publication year, periods of follow-up and all of the adjustable variables (Table 3).

#### Publication bias

The funnel plot (Supplementary Figure 1A), Egger’s test (*P* = 0.511) and Begg’s test (*P* = 0.452) did not suggest significant evidence of publication bias. The sensitivity analysis suggested that the change in recalculated RRs was not significant, with a range from 0.87 (0.73–1.05) when excluding Inoue-Choi 2011 to 0.95 (0.80–1.12) when excluding Shigihara 2014.

#### Subgroup analysis according to gender

Two studies with men and 2 studies with women were included. The results (Table 4, Figure 2B, Figure 2C) indicated that fruit and vegetable consumption is not associated with PC risk in men (RR = 0.87, 95% CI = 0.57–1.33) without heterogeneity (*P* = 0.29, *I*^*2*^ = 12%) and in women (RR = 0.86, 95% CI = 0.43–1.75) with heterogeneity (*P* = 0.07, *I*^*2*^ = 70%).

**Table 4:** Subgroup analyses of pancreatic cancer risk according to gender

Subgroups	*n*	OR (95% CI)	*P*_o_	*P*_h_	*I*^2^ (%)
**Fruits and vegetables** **Male**	2	0.87 (0.57–1.33)	.52	.29	12
**Female**	2	0.86 (0.43–1.75)	.68	.07	70
**Fruits**					
**Male**	3	0.80 (0.66–0.98)	.03	.50	0
**Female**	3	0.99 (0.72–1.36)	.95	.21	35
**Vegetables**					
**Male**	4	0.86 (0.70–1.07)	.18	.28	22
**Female**	4	0.89 (0.71–1.13)	.34	.33	12

*P*_o_: test for over effect. *P*_h_: *P* value for heterogeneity between subgroups. *I*^*2*^: *I*^*2*^ value for heterogeneity within each subgroup.

### The association between fruits consumption with PC risk

#### Highest vs lowest consumption

**Figure 3:**
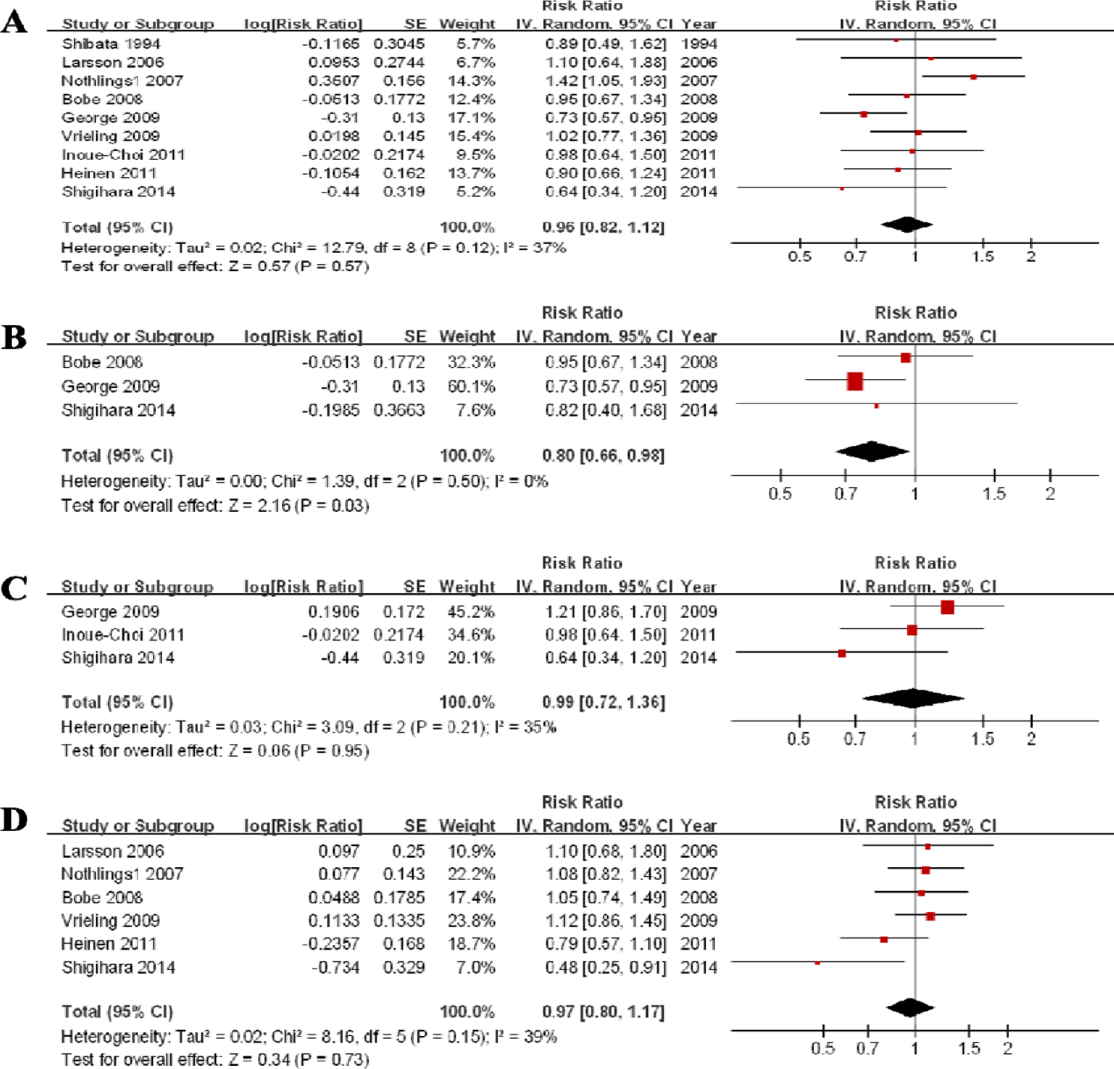
Forest plots of fruits consumption (highest vs lowest category) and PC risk (**A**) Total estimate. (**B**) In men. (**C**) In women. (**D**) for citrus fruit.

Nine studies showed the results for the highest vs lowest consumption. A random-effects model yielded the results (Figure 3A) that fruit consumption was not associated with PC risk (RR = 0.96, 95% CI = 0.82–1.12, *P* = 0.57) without heterogeneity (*P* = 0.12, *I*^*2*^ = 37%) (Table 3).

#### Dose-response analysis

Eight studies were eligible, and the RR per 100 g/d increase in fruit consumption was 1.01 (0.97–1.05) without heterogeneity (*P* = 0.47, *I*^*2*^ = 0%). We further checked for nonlinearity of the dose-response relationship, and there was no evidence of a potential nonlinear relationship between fruit consumption and PC risk (*P*_nonlinearity_ = 0.15).

#### Heterogeneity

There was no significant heterogeneity (*P* = 0.12, *I*^*2*^ = 37%) of the included studies. Subgroup analyses showed that the differences in the RRs were not significant for the geographic area, sample size, publication year, periods of follow-up and all of the adjustable variables except for alcohol (RR = 0.81, 95% CI = 0.71–0.99) (Table 3).

#### Publication bias

The funnel plot (Supplementary Figure 1B), Egger’s test (*P* = 0.911) and Begg’s test (*P* = 0.917) did not suggest significant evidence of publication bias. The sensitivity analysis suggested that the change in recalculated RRs was not significant, with a range from 0.89 (0.78–1.01) when excluding Nothling 2007 to 1.02 (0.89–1.08) when excluding George 2009.

#### Subgroup analysis according to gender

Three studies for men and 3 studies for women were included. The results (Table 4, Figure 3B, Figure 3C) indicated that fruit consumption is not associated with PC risk in women (RR = 0.99, 95% CI = 0.72–1.36) without significant heterogeneity (*P* = 0.21, *I*^*2*^ = 35%). An RR of 0.80 (0.66–0.98) suggested a significant association between fruit consumption and PC risk in men without heterogeneity (*P* = 0.50, *I*^*2*^ = 0%).

#### Subtype analysis for citrus fruit

Six studies were eligible and, the RR was 0.97 (0.80–1.17) without significant heterogeneity (*P* = 0.15, *I*^*2*^ = 39%) (Figure 3D). The sensitivity analysis suggested no significant change in the recalculated RRs, with a range from 0.92 (0.73–1.16) when excluding Vrieling 2009 to 1.03 (0.89–1.18) when excluding Shigihara 2014. The funnel plot (Supplementary Figure 1C), Egger’s test (*P* = 0.151) and Begg’s test (*P* = 0.133) suggested no significant evidence of publication bias.

### The association between fruits consumption with PC risk

#### Highest vs lowest consumption

**Figure 4:**
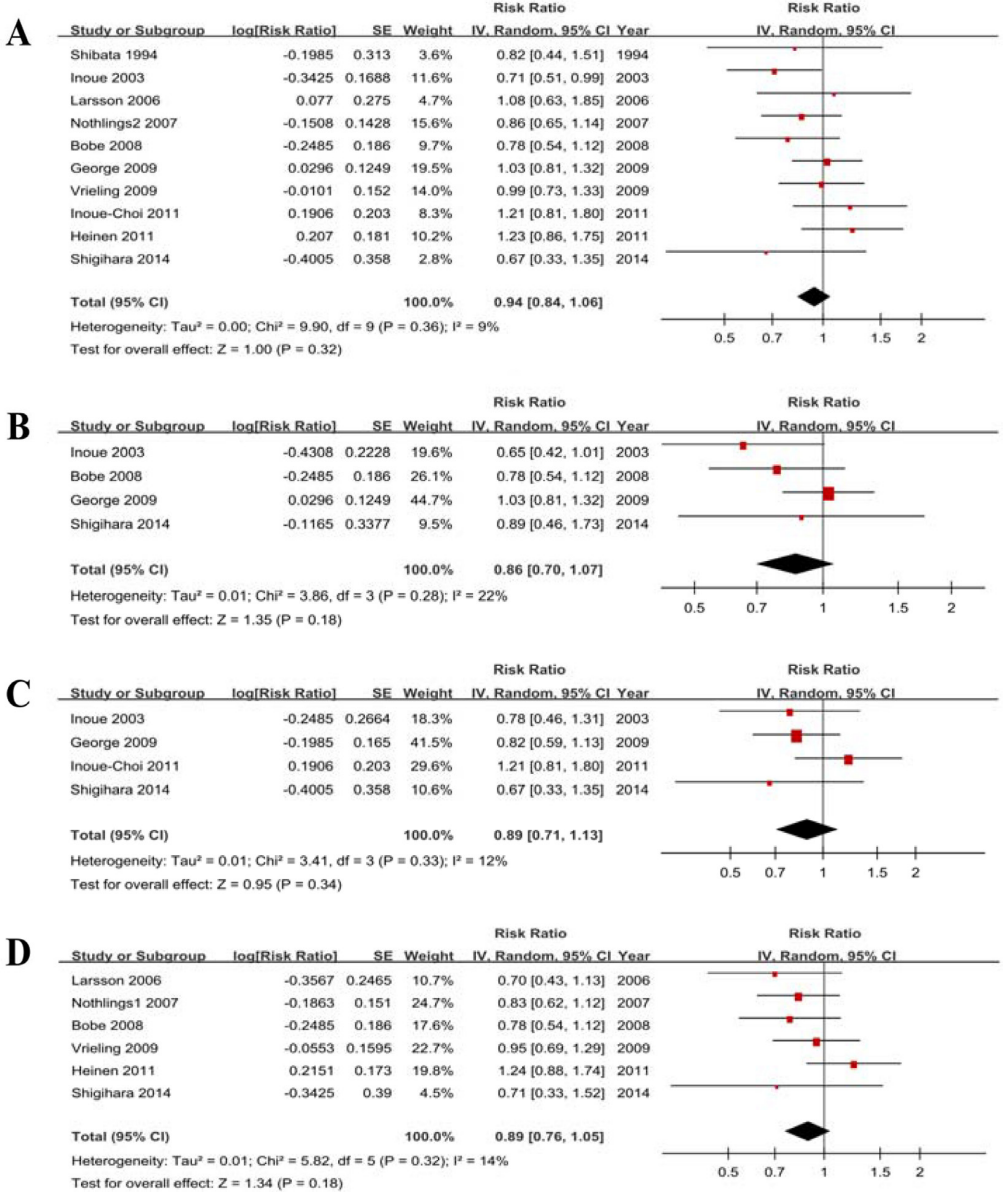
Forest plots of vegetables consumption (highest vs lowest category) and PC risk (**A**) Total estimate. (**B**) In men. (**C**) In women. (**D**) for cruciferous vegetables.

Ten studies showed the results for the highest vs lowest consumption. A random-effects model yielded the results that vegetable consumption was not associated with PC risk (RR = 0.94, 95% CI = 0.84–1.06, *P* = 0.32) without heterogeneity (*P* = 0.36, *I*^*2*^ = 9%) (Figure 4A, Table 3).

#### Dose-response analysis

**Figure 5:**
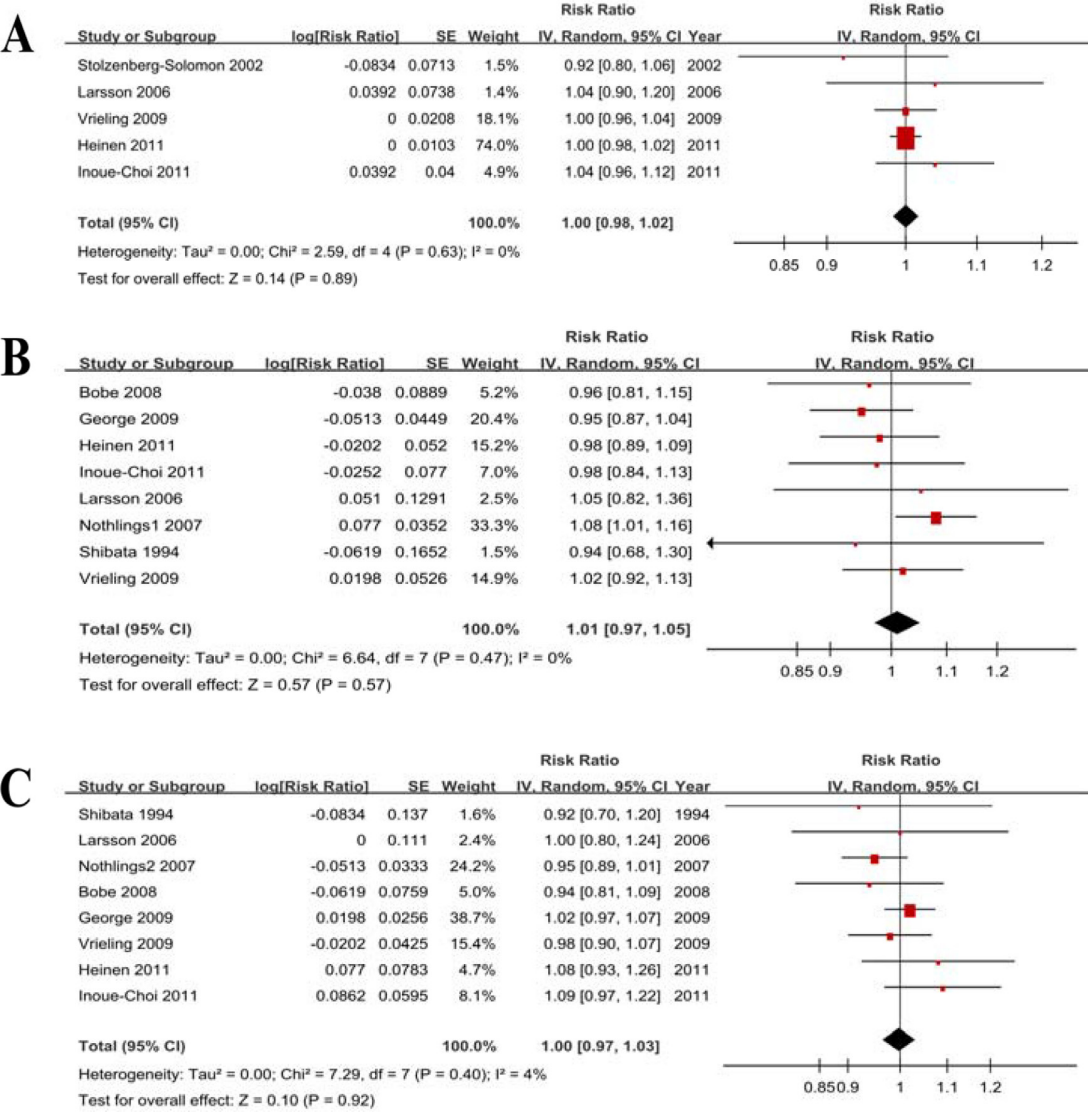
Forest plots of dose-response analyses (**A**) Fruits and vegetables. (**B**) Fruit. (**C**) Vegetables.

Eight studies were eligible, and the RR per 100 g/d increase in vegetable consumption was 1.00 (0.97–1.03) without heterogeneity (*P* = 0.40, *I*^*2*^ = 4%) (Figure 5). We further checked for nonlinearity of the dose-response relationship between vegetable consumption and PC risk, and there was no evidence of a potential nonlinear relationship (*P*_nonlinearity_ = 0.76).

#### Heterogeneity

There was no heterogeneity (*P* = 0.36, *I*^*2*^ = 9%) of the included studies. Subgroup analyses showed that the differences in the RRs were not significant for geographic area, sample size, publication year, periods of follow-up and all of the adjustable variables (Table 3).

#### Publication bias

The funnel plot (Supplementary Figure 1D), Egger’s test (*P* = 0.649) and Begg’s test (*P* = 0.721) did not suggest significant evidence of publication bias. The sensitivity analysis suggested that the change in the recalculated RRs was not significant, with a range from 0.91 (0.81–1.03) when excluding Heinen 2011 to 0.98 (0.87–1.10) when excluding Inoue 2003.

#### Subgroup analysis according to gender

Four studies for men and 4 studies for women were included. The results (Table 4, Figure 4B, Figure 4C) indicated that vegetable consumption is not associated with PC risk in men (RR = 0.86, 95% CI = 0.70–1.07) without heterogeneity (*P* = 0.28, *I*^*2*^ = 22%) and in women (RR = 0.89, 95% CI = 0.71–1.13) without heterogeneity (*P* = 0.33, *I*^*2*^ = 12%).

#### Subtype analysis for cruciferous vegetable

Six studies were eligible, and the RR was 0.89 (0.76–1.05) without heterogeneity (*P* = 0.32, *I*^*2*^ = 14%) (Figure 4D). The sensitivity analysis suggested a significant change in the recalculated RRs, with a range from 0.83 (0.70–0.98) when excluding Heinen 2011 to 0.92 (0.75–1.11) when excluding Bobe 2008. The funnel plot (Supplementary Figure 1E), Egger’s test (*P* = 0.425) and Begg’s test (*P* = 0.452) suggested no significant evidence of publication bias.

## DISCUSSION

In general, fruit and vegetable consumption has been reported to be protective against diabetes [9], cardiovascular disease [10], stroke [11] and some cancers [12, 13]. However, the results were inconsistent in epidemiological studies [14]. Although many case-control studies have suggested inverse associations with the consumption of both fruits and vegetables, bias is possible in these studies due to recall and selection between cases and controls. This evidence was not supported by many cohort studies [14].

Our findings provide more detailed evidence that high total consumption of fruits and vegetables, fruits, vegetables, citrus fruit or cruciferous vegetables is not associated with a decreased risk of PC overall; although increased consumption of fruits but not vegetables is associated with a lower PC risk in men but not in women, the included studies are limited. Furthermore, dose-response analyses suggested that there are no significant dose-response relationships between a 100 g/d increment in fruit and vegetable consumption and PC risk. Additionally, the results of subgroup analyses and subtype analyses were consistent with the original analyses. Overall, our analyses based on prospective studies showed that there was no evidence of associations between fruit and vegetable consumption and pancreatic cancer risk, and the detailed findings including subgroup analyses and subtype analyses further clarify the associations between fruit and vegetable consumption and PC risk and can be used as a reference for the update of dietary guidelines.

### Study strengths and limitations

Our study had several strengths. The first strength is that a long duration of the follow-up and the large sample size of the included studies provided robust evidence to date and increased the statistical power. Second, included studies were identified from 13 countries in Europe, North America and Asia, which increased the statistical generalizability. Third, detailed subgroup analyses were conducted according to the main potential confounders of the studies and main adjustable variables of PC, including gender. These independent results increased the significant power and provided more detailed data of reference significance for dietary guidelines concerning PC worldwide. Fourth, we performed dose-response analyses in addition to simply performing comparisons of the lowest versus highest categories, which further verified our results. Fifth, we conducted further subtype analyses for the two main types of fruits and vegetables reported: citrus fruit and cruciferous vegetables; these analyses strengthened our findings in the details. Finally, the heterogeneity between the studies was not statistically significant in all of the analyses except the analysis of fruits and vegetables in women. Additionally, publication bias was negligible based on the results of the funnel plots, Egger’s tests, Begg’s tests and sensitivity analyses. These statistical results increased reliability of our data.

Several limitations of this study must be taken into consideration. First, the included studies were observational studies, which were liable to residual confounding and other unmeasured factors. Nevertheless, the main confounders were adjusted in most included studies, and we further conducted subgroup analyses to assess the effects of these confounders, including sex, smoking, alcohol use, body mass index, family history of PC and history of diabetes mellitus. In general, these findings were similar to the summary estimates and were consistent for each of the subgroup analyses except for alcohol and fruit consumption. Nevertheless, storage conditions, production methods, cooking methods and nutrient content might be different among the included studies, and measurement errors to assess dietary consumption can lead to bias; and we cannot thoroughly exclude potential residual confounding.

Second, the exposure ranges from the lowest to highest categories were different among the included studies, which contributed to incomparable results and heterogeneity to some extent. However, we adopted and pooled the RRs for the comparison of the highest versus lowest category, and the dose-response analyses verified the results. Additionally, there was no statistically significant heterogeneity in all of the analyses except the analysis of fruits and vegetables in women.

Third, although we further explored the associations between the consumption of citrus fruit or cruciferous vegetables and PC risk, there were no subtype analyses for other types of fruits and vegetables. Therefore, our findings should not be used to analyze other specified fruits or vegetables. Additionally, because we only focused on PC, the available results should not be used to determine associations with pancreatic intraepithelial neoplasia and pancreatic benign tumors.

Last but not least, the quality of two included studies was not high [15, 16] and the sample size of several studies was not large (< 200 cases) [15–18] despite meeting the eligibility criteria. Nevertheless, the detailed subgroup analyses addressed these issues and showed that the separate estimates were consistent with the overall results.

Last but not least, the dietary information of all of the included studies was limited to middle-aged and older persons. The average ages were more than 50 years old in all of the studies, and most of the subjects were older than 60 years of age. Our findings cannot capture the pertinent exposure period of whether fruit and vegetable consumption during childhood, adolescence or early adulthood may be protective against PC.

## MATERIALS AND METHODS

### Selection criteria

1. Prospective cohort studies and nested case-control studies were included.
2. Histological features that were not consistent with the diagnostic gold standard of PC such as pancreatic benign tumors and pancreatitis were excluded.
3. Systematic reviews, meta-analyses, narrative reviews, pooled analyses were excluded.
4. Comments, editorials, letters, case reports were excluded.
5. Studies in which only the abstract could be obtained were excluded.
6. Data that could not be combined or that were incomplete were excluded.
7. Studies were limited to those involving humans; and the publication language of the included studies was limited to English.
8. The highest quality studies, the largest sample sizes and the most recent studies when identifying studies with the same patient cohort were selected.

### Search strategy

We comprehensively identified eligible studies through searching the PubMed and EMBASE databases up to March 2017 using the following search terms: *“fruit”, “fruits”, “vegetable”, “vegetables”, “diet”, “dietary”, “food” and “foods”* in combination with *“gastrointestinal/digestive/alimentary/pancreatic/pancreas”*. We identified additional literature by manually searching the reference lists of the reviews and the extracted studies. The two sets of keywords were combined individually. The eligibility criteria were judged independently by two authors (ZZ, PY).

### Study quality

Two researchers (ZZ, PY) independently assessed the study quality using the Newcastle-Ottawa Scale (NOS) [19]. The NOS is judged on three parameters: the elucidation of the exposure or outcomes of interest for case-control or cohort studies, the selection of the study populations and the comparability of the populations. The maximum score was 9 stars, with 7 or more stars indicating a high-quality study [19, 20].

### Data extraction

Two researchers (ZZ, PY) independently extracted primary relevant data from the studies. Disagreement was resolved by reaching a consensus. The first author, country, year of publication, study period, study population, method of dietary assessment, dietary exposure categories, type of dietary exposure measured, adjusted RR (95% CI) (highest to lowest), adjustments and NOS score of each study were summarized and shown in a data extraction sheet (Table 1). For each study, we extracted the RRs and 95% CIs that reflected the greatest degree of control for potential confounders.

### Statistical analysis

Random-effects models were used to quantify the associations between fruit and vegetable consumption and PC risk. The method described by Greenland and Longnecker [21] was used for the dose-response meta-analysis. Only studies that reported the RR with their corresponding 95% CIs for at least three quantitative exposure categories were included. The mean or median level of fruit and vegetable consumption for each category was assigned to the corresponding RR for each study. When the data were not reported, the midpoint of the upper and lower boundaries in each category was assigned as the average consumption. If the lowest category was open-ended, we assumed the lowest boundary to be 0 [22]. When the highest category was open-ended, we assumed the open-ended interval to be the same as that of the adjacent interval [23]. The best-fitting models were used to examine the potential nonlinear dose-response relationships between fruit and vegetable consumption and PC risk [24]. *P*_nonlinearity_ < 0.05 indicates a nonlinear model.

Heterogeneity among the studies was assessed using Cochran *Q* and *I*^2^ statistics according to the updated version 5.1.0 of Cochrane Handbook for Systematic Reviews of Interventions (0%–40% represents minimal to no heterogeneity). Subgroup analyses were conducted to further explore the sources of heterogeneity by publication year, geographic area, sample size, period of follow-up and adjustments (e.g., smoking, alcohol, BMI, family history of PC and history of diabetes).

We used funnel plots, Begg’s test and Egger’s test to assess publication bias (*P* < 0.1 was considered to indicate significant publication bias) [25]. We conducted sensitivity analyses to investigate the influence of a specific study on the pooled risk estimate by removing one study in each turn.

Data were collected and extracted using SPSS 17.0 (Chicago, Illinois, USA). STATA version 12.1 (STATA Corporation, College Station, TX) and RevMan5.3 (The Cochrane Collaboration, Oxford, UK) were used for synthesis and analysis, respectively.

## CONCLUSIONS

This meta-analysis does not support significant associations between fruit and vegetable consumption and pancreatic cancer risk. The observed decrease in PC risk with fruit consumption in men needs further investigation because of limited included studies.

## SUPPLEMENTARY MATERIALS FIGURE


