# Supplementary material for: No associations between fruit and vegetable consumption and pancreatic cancer risk: a meta-analysis of prospective studies
Source: Oncotarget. 2017 Dec 8;9(63):32250–61. doi: 10.18632/oncotarget.23128 (PMC6114958; doi:10.18632/oncotarget.23128)
Supplement: Supplementary file 1 [file oncotarget-09-32250-s001.pdf]

No associations between fruit and vegetable consumption and pancreatic cancer risk: a meta-analysis of prospective studies

SUPPLEMENTARY MATERIALS

A

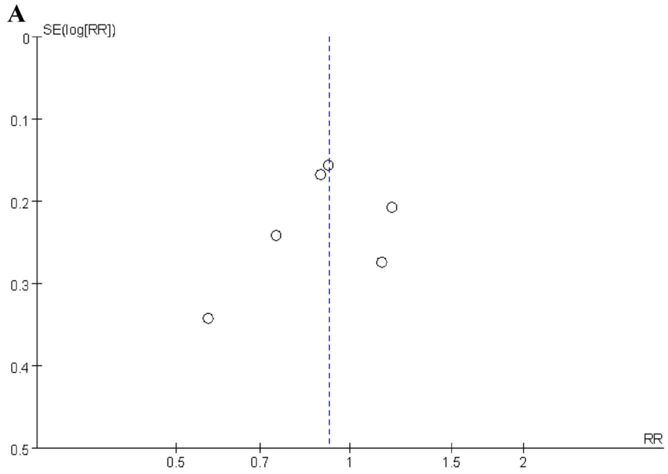

B

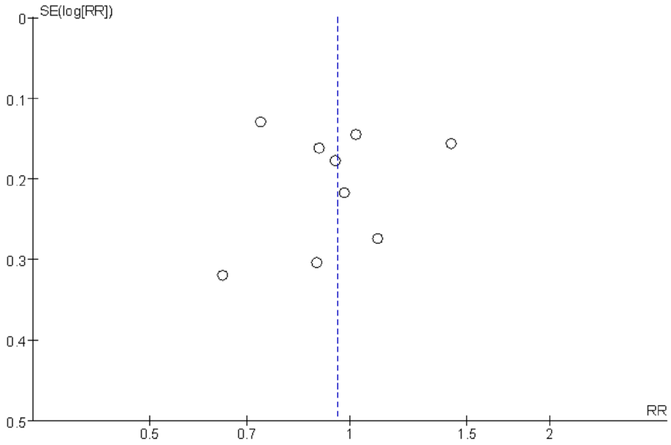

C

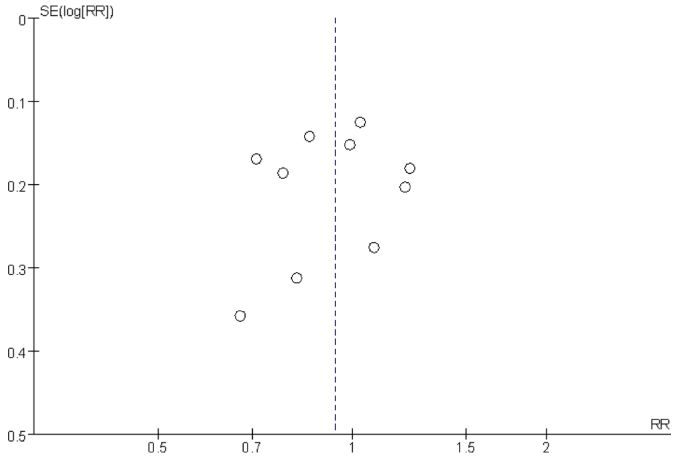

D

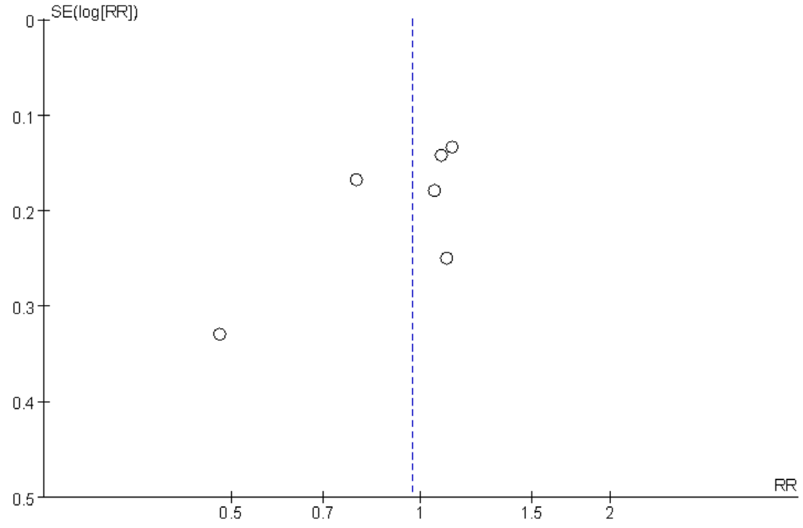

E

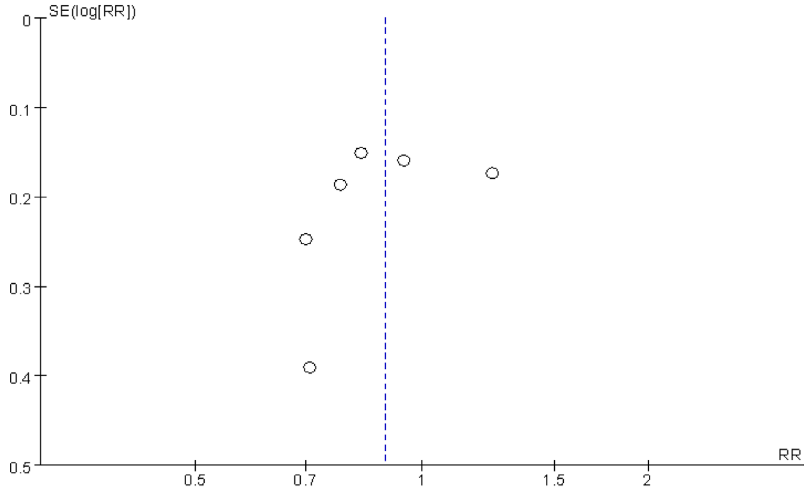

SE: standard error; RR: relative risks.

Supplementary Figure 1: Funnel plots evaluating publication bias of fruits and vegetables consumption and PC risk.
SE: standard error; RR: relative risks. (**A**) fruits and vegetables. (**B**) Fruits. (**C**) Vegetables. (**D**) Citrus fruits. (**E**) Cruciferous vegetables.
